# Oridonin induces apoptosis and senescence in colorectal cancer cells by increasing histone hyperacetylation and regulation of p16, p21, p27 and c-myc

**DOI:** 10.1186/1471-2407-10-610

**Published:** 2010-11-06

**Authors:** Feng-Hou Gao, Xiao-Hui Hu, Wei Li, Hua Liu, Yan-Jie Zhang, Zhu-Ying Guo, Mang-Hua Xu, Shi-Ting Wang, Bin Jiang, Feng Liu, Ying-Zheng Zhao, Yong Fang, Fang-Yuan Chen, Ying-Li Wu

**Affiliations:** 1 NO.3 People's Hospital affiliated to Shanghai Jiao-Tong University School of Medicine (SJTU-SM), Shanghai 201900, PR China; 2 Dept. of Pathophysiology, Key Laboratory of Cell Differentiation and Apoptosis of National Ministry of Education, Shanghai Jiao-Tong University School of Medicine (SJTU-SM), Shanghai 200025, PR China; 3 Zhejiang Provincial Key Laboratory of Medical Genetics, School of Life Sciences, Wenzhou Medical College, Wenzhou, Zhejiang, PR China; 4 Department of Gastroenterology, The tenth Hospital Affiliated to Tongji University, Shanghai, PR China; 5 Renji Hospital affiliated to Shanghai Jiao-Tong University School of Medicine (SJTU-SM), Shanghai 201900, PR China

## Abstract

**Background:**

Oridonin, a tetracycline diterpenoid compound, has the potential antitumor activities. Here, we evaluate the antitumor activity and action mechanisms of oridonin in colorectal cancer.

**Methods:**

Effects of oridonin on cell proliferation were determined by using a CCK-8 Kit. Cell cycle distribution was determined by flow cytometry. Apoptosis was examined by analyzing subdiploid population and terminal deoxynucleotidyl transferase-mediated dUTP nick end labeling assay. Senescent cells were determined by senescence-associated β-galactosidase activity analysis. Semi-quantitative RT-PCR was used to examine the changes of mRNA of p16, p21, p27 and c-myc. The concomitant changes of protein expression were analyzed with Western blot. Expression of AcH3 and AcH4 were examined by immunofluorescence staining and Western blots. Effects of oridonin on colony formation of SW1116 were examined by Soft Agar assay. The in vivo efficacy of oridonin was detected using a xenograft colorectal cancer model in nude mice.

**Results:**

Oridonin induced potent growth inhibition, cell cycle arrest, apoptosis, senescence and colony-forming inhibition in three colorectal cancer cell lines in a dose-dependent manner in vitro. Daily i.p. injection of oridonin (6.25, 12.5 or 25 mg/kg) for 28 days significantly inhibited the growth of SW1116 s.c. xenografts in BABL/C nude mice. With western blot and reverse transcription-PCR, we further showed that the antitumor activities of oridonin correlated with induction of histone (H3 and H4) hyperacetylation, activation of p21, p27 and p16, and suppression of c-myc expression.

**Conclusion:**

Oridonin possesses potent in vitro and in vivo anti-colorectal cancer activities that correlated with induction of histone hyperacetylation and regulation of pathways critical for maintaining growth inhibition and cell cycle arrest. Therefore, oridonin may represent a novel therapeutic option in colorectal cancer treatment.

## Background

Colorectal cancer (CRC) is one of the most frequently diagnosed malignancies in both men and women, with more than 1,000,000 new cases annually worldwide [1]. Advances in therapies over the past decade have led to improved outcomes for many patients. Although curative resection is the major treatment option, approximately half of all patients eventually develop distant metastases. Liver metastases (LM) occur in more than 50% of CRC patients, but curative liver resection is possible only in 15% of them, resulting in 5-year survival rates of 30% on average [2-4]. Improving resectability rates and hopefully patient's prognosis by adding up front active chemotherapy and biological agents in metastatic CRC is a challenging opportunity for both medical and surgical oncologists [5]. Thus, effective new cytotoxic chemotherapy is needed for these diseases.

The terpenoids constitute the largest family of natural products; over 22,000 individual compounds of this class have been described, and the number of defined structures has doubled every decade since the 1970s [6]. In plants, terpenoids represent a chemical defense against environmental stress and provide a repair mechanism for wounds and injuries. Interestingly, effective ingredients in several plant-derived medicinal extracts are also terpenoid compounds of monoterpenoid, sesquiterpenoid, diterpenoid, triterpenoid and carotenoid groups. Experimental study shows that many of them have strong anti-tumor activity [7]. For example, Tanshinone IIA, the major active diterpene quinine in the herbal product from the roots of Salvia miltiorrhiza, is a commonly used Chinese plant remedy which seems to have some activity against breast cancer [8,9]. Celastrol, a quinone methide triterpenoid, isolated from the Chinese Thunder of God Vine (Tripterygium wilfordii Hook F.), as well as triptolide, are currently being investigated in the search for prevention of tumor cell invasion [10]. Plant-derived terpenoids provide a challenging field to identify new potent natural anticancer compound for the therapy of colorectal cancer.

Oridonin, an ent-kaurane diterpenoid isolated from Rabdosia rubescens, is an important traditional Chinese herbal remedy. Studies showed that oridonin induced apoptosis in a variety of cancer cells including those from prostate, breast, non-small cell lung cancer, acute leukemia, glioblastoma multiforme and human melanoma cells. Cell culture experiments have indicated that oridonin inhibits cell cycle progression and induces apoptosis as well as enhance the phagocytosis of apoptotic cells by macrophages [11,12]. Oridonin has also immunosuppressive properties both in vitro and in vivo [13]. However, mechanisms underlying the antitumor activity of oridonin and whether oridonin has the anti-colorectal cancer activity remain largely unknown.

In this report, we found that oridonin could induce potent growth inhibition, cell cycle arrest, apoptosis and senescence of colorectal cancer cells in vitro and in vivo. The antitumor activities of oridonin correlated with induction of histone (H3 and H4) hyperacetylation, activation of p21, p27 and p16, and suppression of c-myc expression.

## Methods

### Cell Culture and Reagents

The colorectal cancer cell lines SW1116, HT29 and HCT116 from Shanghai Institutes for Biological Sciences were incubated in humidified room air containing 5% CO_2 _at 37°C and cultured in McCOY'S 5A medium (Sigma, USA) supplemented with 10% fetal bovine serum (FBS) and 1% penicillin-streptomycin (GIBCO BRL, Grand Island,NY). Cells were routinely grown in 100 mm plastic tissue culture dishes (Nunc, Roskilde, Denmark) and harvested with a solution of trypsin-EDTA when they were in logarithmic phase of growth. Cells were maintained at these culture conditions for all experiments. Oridonin (purity > 98%) was purchased from CHENGDU MUST BIO-TECHNOLOGY CO.LTD. It was dissolved in DMSO at a stock concentration of 100 mmol/L and store at -20°C. The stock solution was further diluted with cell culture medium to yield final oridonin concentrations.

### Cell Proliferation Assay

Cells were seeded into 96-well plates at 2,000 to 3,000 live cells per well and treated with Oridonin (6.25-100 μM) for 3 days. The antiproliferative effect of Oridonin was assessed using Cell Count Kit-8 (Dojindo Molecular Technologies, Inc., Gaithersburg, MD).

### Cell Cycle Analysis with Flow Cytometry

Cells treated with or without Oridonin (12.5 and 25 μmol/L) were harvested for flow cytometry analysis on day 1. Cells were fixed and stained with 0.1 mg/mL propidium iodide for DNA analysis with Becton Dickinson FACScan (Franklin Lakes, NJ) as described previously [14].

### Detection of Apoptosis

Apoptosis was evaluated with flow cytometry and on cell smears using the terminal deoxynucleotidyl transferase-mediated dUTP nick end labeling assay (In situ Cell Death Detection kit, AP; Boehringer Mannheim GmbH, Mannheim, Germany). Samples were incubated with 50 μL of reaction mixture in a humidified chamber at 37°C for 90 minutes as described previously [15]. The percentage of apoptotic cells was determined by counting at least 1,000 cells from 10 to 20 high-power fields (×200) under both phase-contrast and fluorescent microscopy.

### Cell Senescence Assay

Senescence-associated expression of β-galactosidase activity [16] was done with a Senescence Detection kit (BioVision, Mountain View, CA) on fixed cells treated with or without Oridonin (12.5 and 25 μmol/L). The development of cytoplasmic blue was detected and photographed using a Nikon (Nikon Instruments, Inc., Lewisville, TX) inverted microscope equipped with a color CCD camera.

### RNA Extraction and Semi-quantitative RT-PCR

Total RNA was extracted from cell cultures using TRI REAGENT (Molecular Research Center, Inc., OH) according to the manufacturer's protocol. The mRNA levels of the genes analyzed were measured by RT-PCR amplification. Sequences for mRNAs from the nucleotide data bank (National Center for Biotechnology Information) were used to design primer pairs for RT-PCR reactions (Primer Express, Applied Biosystems, CA). The following specific oligonucleotide primers were used respectively for p16 (p16-F: 5'-CAC GGC CGC GGC CCG GGG TC -3' and p16-R: 5'-GGC CCG GTG CAG CAC CAC CA -3' ), p21(p21-F: 5'-AGG CGC CAT GTC AGA ACC GGC TGG -3' and p21-R: 5'-GGA AGG TAG AGC TTG GGC AGG C-3' ), p27 (p27-F: 5'-ATG TCA AAC GTG CGA GTG TCT AAC -3' and p27-R: 5'-TTA CGT TTG ACG TCT TCT GAG GCC A-3' ), c-myc (c-myc-F: 5'-ATT CTC TGC TCT CCT CGA -3' and c-myc-R: 5'-TCT TGG CAG CAG GAT AGT -3' ) with GAPDH as internal control (GAPDH-F: 5'-TCC CAT CAC CAT CTT CCA G-3' and GAPDH-R:5'-ATG AGT CCT TCC ACG ATA CC-3';). PCR cycles were adjusted to have linear amplification for all the targets. Each RT-PCR reaction was repeated at least three times. A semiquantitative analysis of mRNA levels was carried out by the ''GEL DOC UV SYSTEM'' (Biorad Company, CA).

### Western-blot analysis

Attached cells were collected by scraping them off into a lysis buffer, and the detached cells in the supernatant were collected by centrifugation before resuspension in the lysis buffer. Protein concentration was determined by the bicinchoninic acid (BCA) method according to the manufacturer's (Pierce, Rockford, IL, U.S.A.) instructions after trichloroacetic acid precipitation. The protein lysates were mixed with equal volume of Laemmli buffer (62.5 mM Tris-HCl pH 6.8, 2% SDS, 50 mM DTT, 10% glycerol, 0.01% bromophenol blue), boiled for 3 min at 100°C, and then resolved by SDS-PAGE on a 10 to 12% gel using a mini gel apparatus (Bio-Rad). Bromophenol Blue (0.01%) was added to the samples before an equal amount of proteins was loaded in each lane for electrophoresis and blotting. The PVDF membrane was incubated with a primary antibody against cdc2, cdc25c and cyclinB (Santa Cruz Biotechnology), p16 (PharMingen, San Diego, CA, U.S.A.), p21 (PharMingen, San Diego, CA, U.S.A.), p27 (PharMingen, San Diego, CA, U.S.A.), c-Myc (N-262; Santa Cruz Biotechnology, Santa Cruz, CA, U.S.A.), Acetylated histone H3 (AcH3) and H4 (AcH4), phospho-Histone H3 (Ser10) (Upstate Biotechnology, Lake Placid, NY), histone 3, histone 4 and GAPDH (Santa Cruz Biotechnology) for 2 h. Signals were detected using a horseradish peroxidase-conjugated secondary antibody and an enhanced chemiluminescence detection kit (ECL; Amersham Biosciences, Pittsburgh, PA) [17] and were quantitated by an Eagle Eye II Image System with installed density-analysis software (Stratagene, La Jolla, CA, U.S.A.).

### Immunofluorescent Staining

Cells were fixed with 4% paraformaldehyde in PBS for 15 min at room temperature for anti-acetyl histone H4 (06-598; Upstate Biotechnology) and anti-acetyl histone H3 (06-599; Upstate Biotechnology) staining. Cells were permeabilized with 0.2% Triton X-100 (EM Science, Gibbstown, NJ) in PBS for 10 min at room temperature. FITC-labeled secondary antibody (F-0382; Sigma) were applied at the concentration of 1:500. Images were taken with Nikon E800 scope. Senescence-associated heterochromatin staining was conducted as described [18].

### CFE in Soft Agar

Tumor cells were resuspended in DMEM with 0.3% agar and plated in 24-well plates at 2,000 per well on top of a 0.5 mL precast semisolid 1% agar underlayer following treatment with Oridonin ( 0, 6.25, 12.5, 25, 50 or 100 μmol/L) for 2 weeks as described previously [15]. The CFE was defined as the percentage of plated cells that formed colonies relative to an untreated control.

### Murine model and oridonin treatment

Five-week-old pathogen-free athymic nude mice were purchased from Experimental Animal Centre of SIBS. (Shanghai, PR China). BALB/C nude mice were bred and maintained in a specific pathogen-free environment. Mice were allowed free access to mice standard food pellets and tapwater. Twice a week cages were cleaned and water changed. Temperature was controlled at 21°C ± 2°C. The light was on a 12 hours light-12 hour dark cycle, with light on at 8 am. Xenograft model in nude mice was established by subcutaneous inoculation of 1 × 10^6 ^SW1116 cells into the right flank. The nude mice received oridonin treatment (6.25, 12.5 or 25 mg/kg per day) when tumor was measurable. Caliper measurements of the longest perpendicular tumor diameters were performed every day to estimate the tumor volume, using the following formula: 4π/3 × (width/2)^2 ^× (length/2), representing the 3-dimensional volume of an ellipse [19]. Animals were killed when their tumors reached 2 cm or when the mice became moribund. TUNEL assay was performed to detect in situ apoptosis on tissue section using a DeadEnd Colorimetric TUNEL System (Promega) according to the manufacturer's instructions. Senescence-associated expression of β-galactosidase activity [20] was detected with a Senescence Detection kit (BioVision, Mountain View, CA) in situ senescence on tissue section. Animal related experiments were performed according to the Guide for the Care and Use of Laboratory Animals (NIH Publications No. 80-23, revised 1996) and approved by the committee for human treatment of animals at Shanghai Jiao Tong University School of Medicine.

### Statistical Analysis

The effects of oridonin on cell proliferation, CFE, cell cycle arrest, apoptosis, and xenograft growth in SCID mice were analyzed with two-way ANOVA and presented as the mean ± SD.

## Results

### Oridonin suppresses colorectal cell proliferation

**Figure 1:**
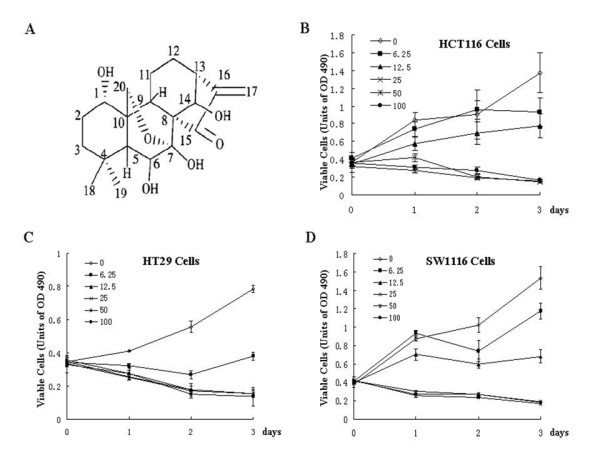
**Oridonin inhibits cell proliferation of colorectal cancer cells**. (A) Chemical structure of oridonin. (B), (C) and (D) HCT116, HT29 and SW1116 cells were treated with 0, 6.25, 12.5, 25, 50 100 μM oridonin for 1, 2 and 3 days. Effects of oridonin on cell proliferation were determined by using a CCK-8 Kit. Error bars represent SD of experiments.

To investigate the possible effect of oridonin on the proliferation of colorectal cancer cells, three colorectal cancer cell lines HCT116, HT29, SW1116 were used. As shown in Figure 1, oridonin could inhibit proliferation of the three colorectal cancer cells in a time- and dose-dependent manner. These tumor cells showed different sensitivity to the oridonin treatment. It seemed that HT29 cells are more sensitive to oridonin treatment than HCT116 and SW1116 cells. The growth of HT29 cells was greatly inhibited by oridonin at 6.25 μM for 24 hours, which become more obvious on day 3. However, 12.5 μM oridonin was needed to obtain 50% inhibition in HCT1116 and SW1116 cells on day 3. At 25 μM, growth of all three colorectal cancer cell lines was completely inhibited.

### Oridonin induces cell cycle arrest and augments apoptosis

**Figure 2:**
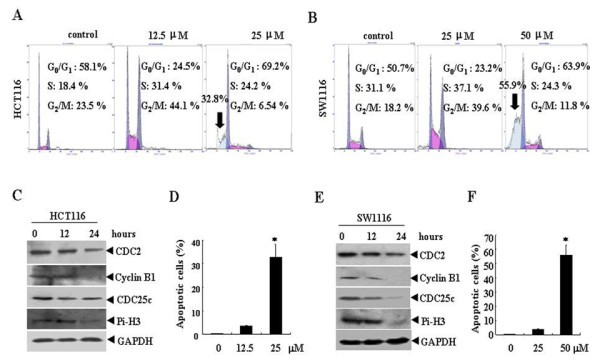
**Oridonin induces cell cycle arrest and apoptosis**. HCT116 and SW1116 cells were treated with 0, 12.5, 25 or 50 μM Oridonin for 24 hours. Cell cycle distribution was determined by flow cytometry and the representative graphs are shown in (A) and (B). Apoptosis was examined by analyzing subdiploid population (A and B, black arrow) and terminal deoxynucleotidyl transferase-mediated dUTP nick end labeling assay (D and F), in which at least 1,000 cells from randomly selected high-power fields were counted. Symbol * represents P < 0.05 compared with control group. The indicated proteins were detected by western blot (C and E).

We next examined the effects of oridonin on cell cycle distribution and apoptosis. HCT116 and SW1116 cells were treated with oridonin at low dose (12.5 μM for HCT116 or 25 μM for SW1116) or at higher dose (25 μM for HCT116 or 50 μM for SW1116) for 24 hours. Low dose oridonin causes obvious G2/M arrest in HCT116 and SW1116 cells. As shown in Figure 2A and 2B, the population of G2/M cells increased from 23.5% and 18.2% to 44.1% and 39.6%, respectively in HCT116 and SW1116 cells. Further, the G2/M related protein cyclin B, cdc2, cdc25c and phosphorylated histone H3 were detected in HCT116 and SW1116 cells (Figure 2C and 2E). Oridonin treatment for 12, 24 h causes down-regulation of all these proteins, indicating that oridonin induces G2 arrest in these cells.

While at higher concentration (25 μM for HCT116 or 50 μM for SW1116), a marked increase of subdiploid peak was observed, indicating the increase of apoptotic cells. This apoptosis inducing effect of oridonin was further confirmed by the terminal deoxynucleotidyl transferase-mediated dUTP nick end labeling assay (Figure 2D and 2F). These results suggested cell cycle arrest and apoptosis induction involved in oridonin induced cell proliferation inhibition.

### Oridonin induces cellular senescence

**Figure 3:**
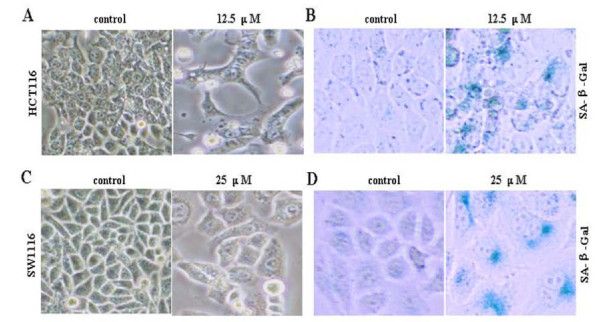
**Oridonin induces cellular senescence**. HCT116 and SW1116 cells were treated with 0, 12.5 or 25 μM oridonin for 72 hours. Morphology of control cells and oridonin-treated cells were examined by phase-contrast microscopy (A and C) (×200). Senescent cells were determined by senescence-associated β-galactosidase activity analysis (B and D) (×200).

Cellular senescence has been identified as one of the mechanisms mediating the anticancer effects of chemotherapies [16]. One of the morphologic changes that were frequently observed in the oridonin-treated cells is the flattening of the adherent cells with increased granularity, which is a typical morphologic change associated with cellular senescence (Figure 3A and 3C). By examining senescence-associated expression of β-galactosidase activity, we confirmed that cellular senescence was indeed induced in those flattened HCT116 and SW1116 cells treated by oridonin (12.5 or 25 μM) for three days (Figure 3B and 3D), indicating that senescence inducing also contribute to the proliferation inhibition effect of oridonin.

### Oridonin activates p21, p16 and p27 expression and down-regulates the expression of oncogene c-myc

**Figure 4:**
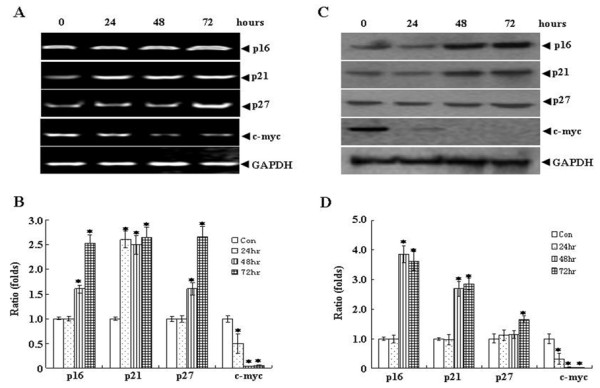
**Oridonin regulates the expression of p21, p16, p27 and c-myc in vitro**. SW1116 cells were treated with 25 μM Oridonin for 0, 24, 48 and 72 hours. Semi-quantitative RT-PCR was used to examine the changes of mRNA of p16, p21, p27 and c-myc (A). (B) The graph shows densitometric analyses of the p16, p21, p27 or c-myc/GAPDH the ratios from (A). The concomitant changes of protein expression were analyzed with Western blot (C). (D) The graph shows densitometric analysis of the p16, p21, p27 or c-myc relative to GAPDH. Symbol * represents P < 0.05 compared with control group.

To perform detailed temporal analysis of gene expression alteration, SW1116 cells were treated with 25 μM oridonin for 0, 24, 48 and 72 hours. Then, the mRNA and protein levels of p16, p21, p27 were examined. As shown in Figure 4A and 4C, after treated oridonin for 24 hours, the mRNA and protein level of p21 but not p16 and p27 were significantly increased. Treatment with oridonin for 48 and 72 hours resulted in increase of the p16, p21 and p27. A series of studies have documented that c-myc regulates a wide range of genes involved in cell proliferation, differentiation, and apoptosis [21]. We also detected the expression of c-myc in oridonin treated SW1116 cells. Treatment with oridonin (25 μM) dramatically reduced c-myc mRNA transcription and c-myc protein levels as early as 24 hours. These data suggested that suppression of c-myc and up-regulation of p16, p21 and p27 correlated with oridonin responsiveness of colorectal cancer cells.

### Oridonin induces global changes in chromatin structure

**Figure 5:**
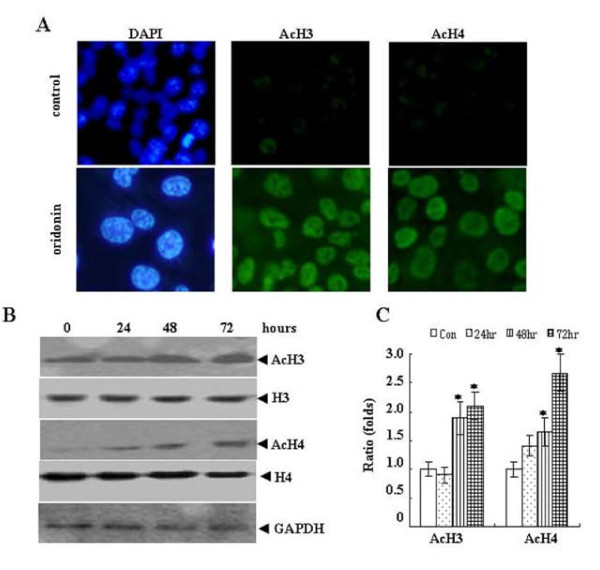
**Induction of hyperacetylated histone H3 (AcH3) and H4 (AcH4) during the treatment with oridonin**. SW1116 cells were treated with 25 μM oridonin for 72 hours. (*A*) Heterochromatin formation was shown by DAPI staining. Distribution of AcH3 and AcH4 were examined by immunofluorescence staining with the indicated antibodies (×200). (B) Protein levels of AcH3 and AcH4 were detected by Western blots with the indicated antibodies. H3 , H4 and GAPDH were used as loading control. (C) The graph shows densitometric analysis of the expression of AcH3 and AcH4 relative to GAPDH. Symbol * represents P < 0.05 compared with control group.

Cellular senescence is known to be associated with changes in chromatin structure [18,22]. Therefore, we examined global chromatin modifications associated with cellular senescence in SW1116 cell lines treated with oridonin (25 μM) for 3 days *in vitro*. As shown in Figure 5A, after 72 h of oridonin treatment, heterochromatin formation were observed with DAPI staining, and a marked global increase of total acetylation of histone H3 and histone H4 were detected by immunofluorescence. Time course analysis showed that protein levels of acetylation of histone H3 and histone H4 gradually increased, especially for histone H4 (Figure 5B and 5C). Hence, oridonin treatment resulted in global changes of histone modifications that have been associated with cellular senescence.

### Oridonin Suppresses CFE

**Figure 6:**
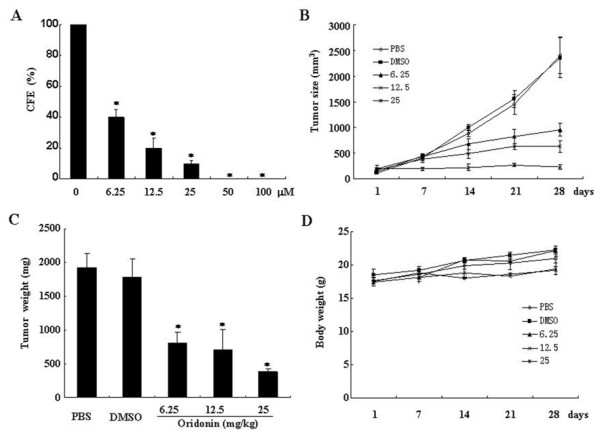
**Oridonin inhibits colony formation of SW1116 in vitro and tumor growth in vivo**. SW1116 cells were treated with 0, 6.25, 12.5, 25, 50, 100 μM oridonin for 14 days. (A) The CFE was defined as the percentage of plated cells that formed colonies relative to an untreated control (untreated cells = 100%). The means ± standard deviations of six independent experiments per oridonin concentration are shown. The symbol * represents P < 0.05 compared with control group. SW1116 cells (1 × 10^7 ^cells in 100 μl of PBS) were subcutaneously injected into the right flank of BALB/c nude mice. The mice were then administered intraperitoneally with 0.2 ml of PBS or DMSO (1% in PBS) or Oridonin (6.25, 12.5 and 25 mg/kg respectively) daily when tumors reached a volume of 50-100 mm^3^. (B) Tumor dimension was periodically measured using calipers over a 4-week period. Each point represents average volume calculated from ten mice. (C) One day after the last treatment, tumors were excised from the animals, and tumor weight was measured. The data are representative of three independent experiments. Symbol * represents P < 0.05 compared with control group. (D) Changes in body weight of mice treated with PBS or DMSO (1% in PBS) or oridonin.

To examine the suppressive effects of oridonin on CFE, SW1116 cells were treated with 0, 6.25, 12.5, 25, 50, 100 μM oridonin for 14 days. Our results showed that oridonin exerted dose-dependant suppression of CFE in SW1116 cells (P < 0.01; Figure 6A). Treatment with 6.25 μM oridonin for 2 week causes over 50% inhibition of CFE. With the increase in drug concentration, more significant suppressive effects were observed. At higher concentration (50 and 100 μM), the formation of colony was completely inhibited (Figure 6A).

### Oridonin Suppresses the Growth of SW1116 Xenografts in BALB/C nude mice

**Figure 7:**
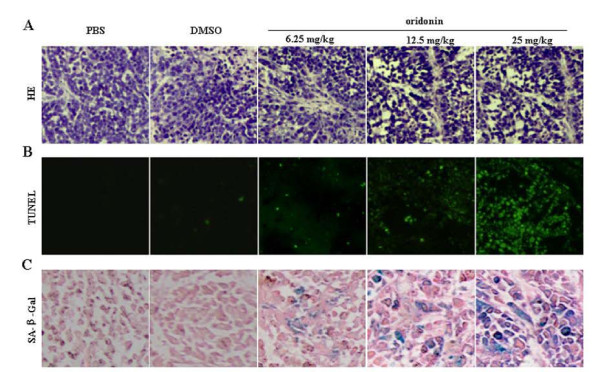
**Oridonin inhibits tumor growth in nude mice through induction apoptosis and senescence**. The mice were administered intraperitoneally with 0.2 ml of PBS or DMSO (1% in PBS) or indicated dose of oridonin for 28 days, then the tumors were excised for pathological examination. (A). Tumor section from PBS or DMSO (1% in PBS) or oridonin treated mice were stained with hematoxylin-eosin (HE) (×200). (B). Apoptotic colorectal cells were assessed by fluorescent TUNEL assay (×100). (C). Senescent colorectal cells were examined by Senescence-associated-β-galactosidase (SA-β-Gal) staining (×200).

We next further assessed the in vivo anti-colorectal cancer effects of oridonin. BALB/C nude mice xenografts were treated with oridonin by daily i.p injection at 6.25 or 12.5 or 25 mg/kg for up to 28 days. Such treatment resulted in significant suppression of xenograft growth , compared with the PBS or DMSO (1% in PBS) treated group (P < 0.05; Figure 6B and 6C). In the low-dose (6.25 mg/kg) group, significant tumor growth inhibition effect was observed until 21 days, while in the middle dose (12.5 mg/kg) group, significant tumor growth inhibition effect was observed on day 14. More interesting, the high dose (25 mg/kg) administration almost completely inhibited tumor growth at the onset phase. It is worth noting that no obvious changes in body weight were observed in the oridonin treated groups, compared with the PBS or DMSO (1% in PBS) treated groups (Figure 6D). In H & E stained tumor section, sparse tumor cells and areas of necrosis foci can be seen in oridonin treated mice, while massive tumor cells were observed in PBS or DMSO (1% in PBS) treated mice (Figure 7A). TUNEL assay (Figure 7B) and Senescence-associated-β-galactosidase (SA-β-Gal) staining (Figure 7C) were also performed to detect the degree of apoptosis and senescence. The results showed that oridonin induces apoptosis and senescence of colorectal cells in vivo in a dose dependent manner.

## Discussion

Chemotherapy remains the primary treatment for systemic malignancies. It is now clear that drug-induced damage is not invariably lethal, but can instead initiate a series of post-damage responses including apoptosis, mitotic catastrophe, and cellular senescence [23,24]. Therefore, the integrity of these damage responses might also influence treatment sensitivity. In the current study, we showed that the potent anti-cancer activities of oridonin in colorectal cancer are correlated with induction of apoptosis, cell-cycle arrest and cellular senescence.

Oridonin treatment resulted in significant growth arrest in colorectal cancer cells and this in vitro effect was time and concentration dependent. This finding is in agreement with our previous results with oridonin in acute promyelocytic leukemia cells and others [14]. More importantly, we further showed that oridonin possesses strong inhibitory activities on tumorigenicity of colorectal cancer cells. Oridonin at 6.25 μM could inhibit the colony-forming by 50%. With the increase in drug concentration, the formation of colony was almost completely inhibited. In consistent with these results in vitro, treatment with 6.25 mg/kg oridonin in SW1116 xenografts BALB/C nude mice for 4 weeks was able to significantly decrease the growth of xenografts. These results provided strong evidence to support the notion that oridonin has strong activity against colorectal cancer.

Apoptosis is a well-characterized post-damage program and diverse anticancer agents can induce apoptosis through common pathways [24]. It has been reported previously that the antitumor effect of oridonin was due to its apoptosis induction activity [14,25-28]. In agreement with these reports, our data showed oridonin could induce apoptosis of colorectal cancer cells in vitro and in vivo. In addition to apoptosis induction, we found that oridonin could induce senescence of colorectal cancer cells. In fibroblasts and epithelial cells, senescence is controlled by the p53 and Rb tumor suppressor pathways, although the contribution of each pathway to the program depends on species and cell type [20]. Here, oridonin-induced arrest was accompanied by substantial increases in p21, p16 (an Rb regulator linked exclusively to senescence) and SA-β-gal activity. It is interesting to note that oridonin-induced senescence associated with G2 arrest. In most cases, the senescence is associated with G1/G0 arrest. However, many reports also showed that G2/M arrest is associated with senescence [29-32]. For example, some drugs including DNA damage agent Adriamycin, DNA methylating agent Temozolomide, HDAC inhibitor sodium butyrate could induce G2/M arrest and senescence-like phenotype via p21 induction. Similar to these results, oridonin could also up-regulate the expression of p21, p27 and p16, which may contribute to the observed senescent phenotype.

The expression of cyclin-dependent kinase inhibitor p21 and p27 has been implicated in chemotherapy-induced cell cycle arrest in numerous human cancers [33,34]. In this study, we confirmed that oridonin is capable of activating p21 and p27 gene in colorectal cancer cells. Overexpression of c-myc had been frequently detected in colorectal cancer and is associated with shorter survival and tumor anaplasia [35-37]. C-myc has also been reported to promote cell cycle reentry and proliferation through repression of p21 and p27 expression [38]. Therefore, the suppression of c-myc expression by oridonin may render substantial therapeutic benefits in colorectal cancer patients by inhibiting the driving activities of c-myc in cell proliferation and cell cycle progression. In line with previous reports, our data showed oridonin- induced p21 and p27 and down-regulated c-myc in vitro.

Epigenetic changes are tightly related to neoplastic transformation in colorectal cancers. Histone modifications, recently recognized as a 'histone code' that affects chromatin structure and gene expression also play an important role in the establishment of gene silencing during tumorigenesis. Alterations in histone modifications appear to be primary mediators of epigenetic inheritance in cancer cells [39-41]. The potential reversibility of epigenetic states in the tumor cell is an attractive target for cancer therapy [42]. Our data showed oridonin induced histone (H3 and H4) hyperacetylation in vitro. We also found that the levels of accumulated AcH3 and AcH4 correlated with the degree of in vitro growth suppression in the oridonin-sensitive colorectal cancer cell lines, suggesting that the anti-colorectal cancer effects of oridonin were at least partly mediated through histone H3 and H4 hyperacetylation.

## Conclusions

In summary, we showed that oridonin possesses potent in vitro and in vivo anti-colorectal cancer activities by suppressing cell proliferation, promoting apoptosis, inducing cell cycle arrest and cellular senescence. These results may lay the groundwork for further studies using specific genetically engineered models to establish the causal relationship between oridonin anti-tumor activity and specific genetic pathways and to identify molecular markers that will predict drug responsiveness and guide the development of future clinical therapies.

## Competing interests

The authors declare that they have no competing interests.

## Authors' contributions

F-HG and X-HH carried out the data collection and data analysis, and drafted the manuscript. WL, HL and Y-JZ participated in the design of the study, performed the statistical analysis and participated in the interpretation of data. Z-YG, M-HX and S-TW participated in the design of the study and the acquisition and interpretation of data. BJ, FL, Y-ZZ, YF and F-YC were involved in the interpretation of data and critically revised the manuscript. Y-LW conceived the study, participated in its design and coordination, and helped in statistical analysis and drafting of the manuscript. All authors have read and approved the final manuscript.

## Pre-publication history

The pre-publication history for this paper can be accessed here:

http://www.biomedcentral.com/1471-2407/10/610/prepub
